# Contributions of Fat and Carbohydrate Metabolism to Glucose Homeostasis in Childhood Change With Age and Puberty: A 12-Years Cohort Study (EARLYBIRD 77)

**DOI:** 10.3389/fnut.2020.00139

**Published:** 2020-08-28

**Authors:** Ornella Cominetti, Joanne Hosking, Alison Jeffery, Jonathan Pinkney, Francois-Pierre Martin

**Affiliations:** ^1^Nestlé Institute of Food Safety & Analytical Sciences, Nestlé Research, Lausanne, Switzerland; ^2^Faculty of Medicine and Dentistry, Plymouth University, Plymouth, United Kingdom; ^3^Nestlé Institute of Health Sciences, Nestlé Research, Lausanne, Switzerland

**Keywords:** amino acids, biochemistry, glucose, longitudinal study, phenotyping, substrate oxidation, puberty, type 2 diabetes

## Abstract

Puberty—a period when susceptibility to the onset of Type 2 diabetes (T2D) increases—is marked with profound physiological and metabolic changes. In the EarlyBird cohort, children who developed impaired fasting glycemia in adolescence already exhibited higher fasting blood glucose at 5 years of age, independent of their body mass index (BMI), suggesting that pubertal factors may modify existing predisposition. Understanding how the physiological changes during childhood influence glucose homeostasis and how the central energy metabolism may help deciphering the mechanisms that underlie the risk of developing T2D in children and adults. We investigated these associations by analyzing glycemic variations with molecular markers of central energy metabolism, substrate oxidation status and pubertal stages in the EarlyBird cohort. The EarlyBird study is a non-interventional, prospective cohort study, that recruited 307 healthy UK children at age 5, and followed them annually throughout childhood for 12 years. Longitudinal data on blood biochemistry, respiratory exchange ratio, and anthropometry, available from 150 children were integrated with fasting glycemia. The gradual rise in blood glucose during childhood associates with age-dependent changes in molecular processes and substrate oxidation status, namely (i) greater pre-pubertal fat utilization, ketogenesis, and fatty acid oxidation, and (ii) greater pubertal carbohydrate oxidation and glycolytic metabolism (Cori and Cahill Cycles) associated with different amino acid exchanges between muscle and other tissues (proline, glutamine, alanine). Since children's metabolic and nutritional requirements evolve during childhood, this study has potential clinical implications for the development of nutritional strategies for disease prevention in children.

## Introduction

Diabetes is now one of the most common non-communicable diseases in the world, affecting over 422 million people according to the World Health Organization (WHO) (1). It has been forecasted that one in every three individuals born in the US in the year 2000 will develop diabetes during their lifetime (2). The principal form of diabetes accounting for these projections is Type 2 diabetes (T2D). As a result of the rising prevalence of obesity, T2D is a growing concern in children (3), with puberty being a period of increasing susceptibility to the onset of diabetes (4, 5).

Normal pubertal growth, along with its underlying physiological endocrine changes, affects body composition, muscle mass and strength, and processes including bone development, erythropoiesis, and substrate utilization (6). These key physiological processes are accompanied by changes in biochemical processes, and in turn may influence aerobic and anaerobic fitness (6). Aerobic fitness increases with the development of the cardiovascular and respiratory systems, and skeletal muscle. In addition, anaerobic fitness is influenced by muscle mass, as well as by size-independent factors (e.g., glycolytic metabolism, muscle architecture, neural control), and tends to increase throughout puberty (7). Such complex and rapid changes in biochemical and physiological parameters influence numerous metabolic functions, including total and resting energy expenditure and physical activity. These changes may be important in determining susceptibility to the development of T2D.

Basal metabolism is the energy required for cellular and tissue maintenance. It increases rapidly up to the age of ~2 years, and reaches a plateau in late adolescence when growth velocity and the growth of muscle mass decreases (8). Growth has additional energy requirements to those of basal metabolism, and is unique to this early stage of life. Energy cost for growth has two components; the energy needed for biosynthetic processes in growing tissues and the energy stores deposited in those tissues (e.g., fat and protein) (8). The role of resting energy expenditure and weight gain in children is subject to controversy, with some evidence that lower energy expenditure may be a factor predisposing to childhood obesity (9, 10). During adolescence, the pubertal growth spurt may be associated with a substantial fall in resting energy expenditure, independent of adiposity (11), which may influence long-term body composition and metabolic health outcomes. Food provides macronutrients (carbohydrates, protein, and fats) that are utilized by the body as sources of energy.

The EarlyBird study is a landmark prospective cohort study investigating the origins of T2D in children. This cohort of healthy children has been followed from age 5 to 16 years with annual clinical, anthropometric, and physiological measurements (12). A total of 17% of the initially healthy children in the EarlyBird cohort showed impaired fasting glycemia (IFG) by the age of 15, a risk factor for future diabetes. The children who developed IFG already exhibited higher fasting blood glucose levels at 5 years of age, compared with those who did not subsequently develop IFG, and this effect was independent of BMI (12). Recently, we reported that the occurrence of an early defect in β-cell function among children who go on to develop prediabetes (12), was genetically determined and independent of BMI (13). These studies also revealed how genetic markers are associated with normal glycemic trajectories during childhood. However, prediabetes did not appear until puberty, when insulin resistance was at its highest (12). We successfully applied a longitudinal analysis to explore the metabolite signatures that precede or follow the development of greater levels of insulin resistance (IR) during childhood (14, 15). These analyses provide key insights into metabolite pathways (ketogenesis, fatty acid oxidation, branched-amino acids), with distinct patterns according to chronological age, and different contribution to glucose metabolism (14, 15). In addition, we reported that interpretation of HbA1c for the diagnosis of impaired fasting glycemia was limited due to factors other than glycaemia systematically influencing the variance of HbA1c in youth (16). Understanding how changes in these molecular pathways associate with changes in glucose homeostasis and dietary substrate oxidation in healthy children is necessary to inform the design of preventive measures, such as individualized nutritional recommendations.

The aim of the present study was to determine how temporal glycemic variations during childhood relate to physiological changes in central energy metabolism and substrate oxidation. Therefore, we investigated the associations between individual metabolites, respiratory exchange ratio and fasting glucose concentrations in the EarlyBird cohort, taking into account critical covariates of age, growth, puberty, adiposity, and physical activity.

## Methods

### Study Population

The study was conducted in accordance with the principles of the Declaration of Helsinki II. Ethical approval was granted by the Plymouth Local Research Ethics Committee (1999), and parents gave written consent and children verbal assent. The EarlyBird Diabetes Study incorporates a 1995/1996 birth cohort recruited in 2000/2001 when the children were 5 years old (307 children, 170 boys) (17). Most of the children were white Caucasian and five children out of 307 were of mixed race, reflecting the ethnic mix of the city of Plymouth. According to the pediatric thresholds for overweight and obesity proposed by the International Obesity Task Force (IOTF), 13% of the EarlyBird boys and 26% of girls were overweight at baseline, which included 4 and 5%, respectively who were obese. The thresholds approximate to the 91st and 98th centiles of the 1990 BMI reference curves for the UK, and are deemed to correspond to equivalent thresholds in adulthood. The collection of data from the EarlyBird cohort is composed of clinical and anthropometric variables measured on an annual basis from the age of 5 to 16.

Impaired fasting glycemia was selected as an objective criterion to identify children with an additional risk for future diabetes. For blood metabolic phenotyping, subjects were purposively selected to include children who had shown impaired fasting glucose at one or more time-points during the course of childhood as reported previously (12), and gender-matched normoglycemic children. A total of 150 participants [63 children (44 boys and 19 girls) who had shown IFG by age 16 years and 87 children (61 boys and 26 girls) who had not] were selected. Metabonomics data were available for 130 of these children. Out of the 55 children who had shown impaired fasting glucose in this subset, seven had a first degree relative with T2D or T1D.

### Anthropometrics

Height and weight were measured every 6 months from age 5 years. BMI was derived from direct measurement of height (Leicester Height Measure; Child Growth Foundation, London, U.K.) and weight (Tanita Solar 1632 electronic scales), performed in duplicate and averaged. BMI SD scores were calculated from the British 1990 standards (18). Moderate to vigorous physical activity (MVPA) was measured annually from 5 years by accelerometry (Acti-Graph) (19). Children were asked to wear the accelerometers for 7 consecutive days at each annual follow-up visit, and only recordings that captured at least 4 days (each day incorporating at least 9 h wear time) were used. Pubertal timing was evaluated by means of age at peak height velocity (APHV), determined as the tangential velocity at the middle time point of three consecutive height measurements taken 6 months apart. Chronologic age does not necessarily correlate with physiologic or somatic (i.e., related to the body) pubertal changes (20). The most common way to describe the sequence of changes in secondary sexual characteristics is that published by Marshall and Tanner, commonly referred as Tanner stages (21, 22). The distribution of age at which each tanner stage was reached for boys and girls is provided in Supplementary Tables 1, 2, respectively. There are five Tanner stages. The first stage (Pre-pubertal stage) of puberty begins around 6–8 years of age, long before any physical changes are noticeable. The second stage of puberty usually begins around 9–11 years for girls and 11–13 years for boys. However, it is normal for this to vary by up to 5 years. The second stage marks the beginning of sexual development and physical changes, during which boys and girls experience a large growth spurt. The stages 3 and 4 mark particularly development of secondary sexual characteristics, whilst the stage 5 marks the end of puberty and the staging into the body of an adult. Peripheral blood was collected annually after an overnight fast, blood serum samples were stored at −80°C.

### Laboratory Assessment

The children were fasted overnight for 10 h before venesection. The Homeostatic Model Assessment of Insulin Resistance (HOMA2IR) and the Homeostatic Model Assessment of Beta Cell Function (HOMA2B) were determined each year from fasting glucose (Cobas Integra 700 analyzer; Roche Diagnostics) and insulin (DPC IMMULITE) (cross-reactivity with proinsulin, 1%) using the homeostasis model assessment program, which has been validated in children (23).

### Respiratory Exchange Ratio

Resting energy expenditure was measured by indirect calorimetry using a ventilated flow through hood technique (Gas Exchange Measurement, Nutren Technology Ltd, Manchester, UK). Performance tests reportedly showed a mean error of 0.3 ± 2.0% in the measurement of oxygen consumption and 1.8 ± 1% in that of carbon dioxide production. Measurements were performed in a quiet thermoneutral room (20°C) after an overnight fasting period of at least 6 h, to minimize any effect attributable to the thermic effect of food. Data were collected for a minimum of 10 min and the respiratory exchange ratio was calculated as an indicator for substrate oxidation.

### Blood Metabolic Profiling

Serum samples collected from each child at every age between 5 and 16 years old were subjected to metabonomic analysis. For technical feasibility and to ensure optimal data reproducibility for cohort analysis, a threshold of 1,800 blood serum samples (e.g., 150 different subjects) was determined. Metabolic profiling was carried out by means of proton nuclear magnetic resonance spectroscopy (^1^H NMR) spectroscopy, as reported previously (14, 15). Briefly, 400 μL of blood serum were mixed with 200 μL of deuterated phosphate buffer solution 0.6 M KH_2_PO_4_. ^1^H NMR metabolic profiles of serum samples were acquired with a Bruker Avance III 600 MHz spectrometer equipped with a 5 mm cryoprobe at 310 K (Bruker Biospin, Rheinstetten, Germany) and processed using TOPSPIN (version 2.1, Bruker Biospin, Rheinstetten, Germany) software package. Based on an internal database of reference compounds, representative signals of metabolites were integrated. The signals are expressed in an arbitrary unit corresponding to a peak area normalized to total metabolic profiles. ^1^H NMR spectroscopy being a quantitative method, metabolite peak areas are proportional to metabolite concentrations, and thus their changes are representative of absolute change in metabolite concentrations in the serum. This metabonomics approach targeted the major metabolic pathways, including central energy metabolism, amino acids, carboxylic acids, and lipoproteins and in a highly reproducible manner across more than 1,700 serum samples. In particular, ^1^H-NMR spectroscopy of human blood serum enables the monitoring of signals related to lipoprotein-bound fatty acyl groups found in triglycerides, phospholipids and cholesteryl esters, together with peaks from the glyceryl moiety of triglycerides and the choline head group of phosphatidylcholine.

### Statistical Analysis

Mixed effects modeling was used to assess the association between individual metabolites and fasting glucose, taking into account age, BMI z-scores, and physical activity. Controlling for maturational and growth status is crucial in life course studies, and age at peak height velocity (APHV) is a key measure of maturity that was also taken into account. Random intercepts were included as well as age (categorized to allow for non-linear change in glucose over time), gender, BMI z-score, APHV, MVPA (number of minutes spent in moderate-vigorous physical activity), and individual metabolites (in separate models) as fixed effects. Each metabolite was transformed to a z-score (i.e., standardized with mean of 0 and standard deviation of 1) for analysis. Modeling was carried out in R software (www.R-project.org) using the lmer function in the package lme4 (24) and *p*-values calculated using the Satterthwaite approximation implemented in the lmerTest package (25). Both unadjusted and Bonferroni-adjusted *p*-values are presented. Additional Spearman Correlation analysis was conducted between fasting glucose and serum metabolites, HOMA indexes, HbA1c, and respiratory exchange ratio.

## Results

### Influence of Chronological Age and Pubertal Stage on Population Demographics

Clinical and anthropometric characteristics of the children for the 12-year period are summarized in Table 1 and Supplementary Figure 1. For both genders, there was an increase in fasting glucose throughout childhood, concomitant with increasing BMI-z-score and respiratory exchange ratio, and decreasing physical activity (MVPA). As previously reported, fasting insulin and HOMA-IR decreased until around 8 years, and then increased during puberty until the age of 14 years, before decreasing until the age of 16 years. This pattern was dependent on the time of APHV and BMI z-scores (15).

**Table 1:** Characteristics of the studied Earlybird cohort.

	**Age (years)**	**Boys**	**Girls**
Fasting glucose (mmol/L^−1^)	5	4.3 ± 0.4	4.4 ± 0.4
	6	4.5 ± 0.4	4.4 ± 0.3
	7	4.6 ± 0.5	4.6 ± 0.4
	8	4.8 ± 0.3	4.7 ± 0.4
	9	4.8 ± 0.5	4.9 ± 0.3
	10	4.9 ± 0.3	4.8 ± 0.3
	11	4.8 ± 0.4	4.8 ± 0.3
	12	4.9 ± 0.4	5.1 ± 0.4
	13	5.2 ± 0.3	5.1 ± 0.4
	14	5.2 ± 0.3	5.2 ± 0.5
	15	5.2 ± 0.3	5.2 ± 0.4
	16	5.2 ± 0.3	5.0 ± 0.4
HOMA-IR	5	0.6 ± 0.49	0.81 ± 0.37
	6	0.48 ± 0.44	0.63 ± 0.41
	7	0.37 ± 0.23	0.46 ± 0.20
	8	0.44 ± 0.26	0.60 ± 0.44
	9	0.59 ± 0.39	0.89 ± 0.49
	10	0.88 ± 0.41	1.15 ± 0.71
	11	0.85 ± 0.54	1.19 ± 0.70
	12	0.96 ± 0.56	1.89 ± 1.30
	13	1.10 ± 0.57	1.61 ± 1.00
	14	1.15 ± 0.68	1.57 ± 0.97
	15	0.93 ± 0.56	1.45 ± 1.54
	16	0.83 ± 0.64	0.96 ± 0.84
Fasting insulin (mU/L^−1^)	5	4.3 ± 3.5	5.7 ± 2.6
	6	3.4 ± 3.2	4.4 ± 2.9
	7	2.6 ± 1.5	3.2 ± 1.3
	8	3.0 ± 1.7	4.1 ± 3.0
	9	4.1 ± 2.7	6.1 ± 3.4
	10	6.0 ± 2.9	8.0 ± 5.1
	11	5.8 ± 3.8	8.3 ± 4.9
	12	6.6 ± 3.9	13.1 ± 9.2
	13	7.4 ± 4.0	11 ± 6.9
	14	7.7 ± 4.7	10.7 ± 6.6
	15	6.3 ± 3.7	9.9 ± 10.9
	16	5.6 ± 4.4	6.6 ± 5.9
BMI Z-scores	5	0.22 ± 1.1	0.3 ± 1.23
	6	0.19 ± 0.99	0.58 ± 0.96
	7	0.23 ± 1.06	0.64 ± 0.93
	8	0.2 ± 0.92	0.46 ± 1.04
	9	0.33 ± 1.05	0.57 ± 0.98
	10	0.33 ± 1.04	0.72 ± 1.11
	11	0.33 ± 1.11	0.66 ± 1.16
	12	0.37 ± 1.16	0.75 ± 1.16
	13	0.49 ± 1.21	0.86 ± 1.21
	14	0.33 ± 1.16	0.86 ± 1.17
	15	0.37 ± 1.12	0.89 ± 1.25
	16	0.51 ± 1.13	0.87 ± 1.14
Moderate-vigorous physical activity (min/day)	5	55.1 ± 19.9	46.2 ± 26.6
	6	61.9 ± 23.6	54.8 ± 17.5
	7	63 ± 24.1	52.6 ± 17.2
	8	61.3 ± 23.1	46.7 ± 15
	9	60.9 ± 23.9	40.8 ± 17.3
	10	59.1 ± 26.5	37.2 ± 14.7
	11	57.3 ± 27.4	34.3 ± 20.2
	12	59.6 ± 27.5	35.2 ± 19.6
	13	55.1 ± 23.9	36.4 ± 22.2
	14	49.5 ± 26.6	40.6 ± 30.4
	15	48.3 ± 23.6	31.6 ± 18.1
	16	44.5 ± 23.7	32.1 ± 22.9
Respiratory exchange ratio	5	0.89 ± 0.09	0.92 ± 0.06
	6	0.88 ± 0.08	0.88 ± 0.09
	7	0.90 ± 0.06	0.92 ± 0.08
	8	0.97 ± 0.09	0.99 ± 0.09
	9	0.95 ± 0.1	0.96 ± 0.14
	10	0.95 ± 0.1	0.93 ± 0.1
	11	1.01 ± 0.07	1.01 ± 0.07
	12	0.99 ± 0.06	1.00 ± 0.07
	13	1.00 ± 0.07	1.02 ± 0.09
	14	0.96 ± 0.08	0.96 ± 0.09
	15	0.95 ± 0.3	0.93 ± 0.09
	16	0.95 ± 0.15	0.95 ± 0.09
Age at peak height velocity (years)		13.0 (12.8–13.4)	11.6 (10.8–12.3)

*Age at peak height is reported as median with interquartile range, and other data are reported as mean +/- standard deviation*.

**Figure 1:**
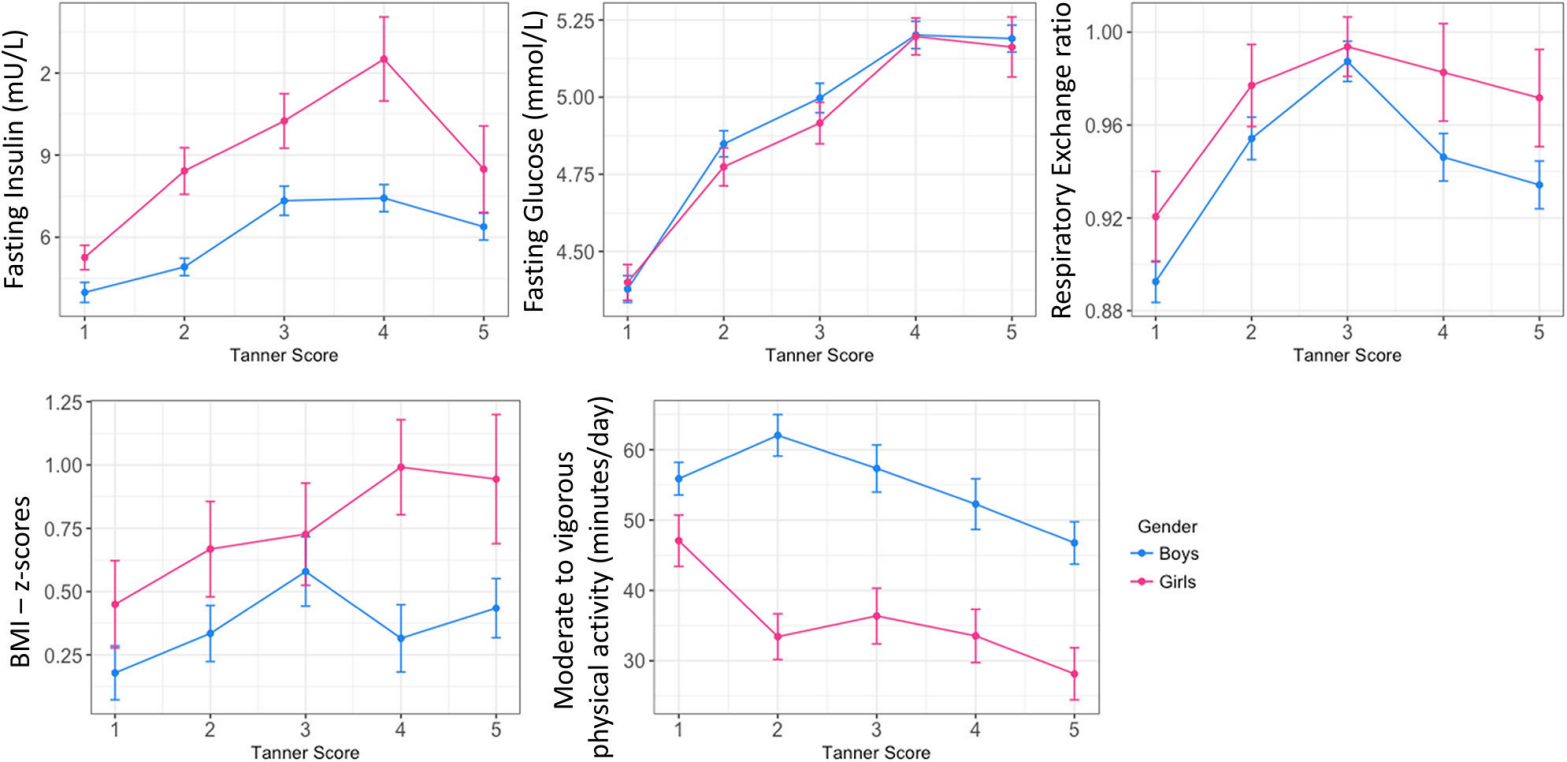
Overview of main glycemic and physiological trajectories in childhood: longitudinal status according to pubertal stage in boys and girls. Data are plotted as mean ± standard error.

Mean fasting glucose concentrations increased from 4.3 and 4.4 mmol at age 5, to 5.2 and 5.0 mmol at age 16, for boys and girls, respectively (Table 1). Interestingly, these increases were marked by two plateaus, first between 8 and 11 years of age, then between 13 and 16 years of age (Supplementary Figure 1). Mirroring the changes in blood glucose concentrations, an evolving pattern was observed in the RER and age. RQ values reflect metabolic substrate utilization for energy production. From age 5 to 7, RER values were around 0.9, and increased toward their maximum values of 1 between the age of 11 and 13, followed by a slight decrease toward 0.95 from 14 years of age (Supplementary Figure 1). Figure 1 describes the age-dependent changes in clinical and glycemic parameters in relation to male and female pubertal development. Parameters were plotted according to Tanner stage (21, 22). Tanner stage was self-reported at each time-point, and the same Tanner stage may be reported at more than one time-point. Therefore, for each child, the parameter for each Tanner stage is represented once by selecting only the value at the first occurrence (e.g., for Tanner Stage 1, values at age 5 were selected).

### Longitudinal Association of Fasting Glucose and Serum Metabolites

Using data at all ages simultaneously, mixed effects modeling was applied to assess the association between fasting glucose concentrations and individual metabolites. Several blood metabolites including amino acids, organic acids, and lipids showed statistically significant associations with fasting glucose concentrations in longitudinal models, independently of BMI z-score, physical activity, and APHV. Data are reported to statistical significance and in alphabetic order for different metabolic pathways and metabolites (Table 2).

**Table 2:** Estimates and *p*-values from mixed effects models examining the association between metabolites and fasting glucose.

**Metabolic pathway**	**Metabolite**	**^1^H NMR chemical shift (ppm)**	**Coef**	**SE**	***p*-value (unadjusted)**	***p*-value (adjusted)**
Alcohol	Methanol	3.31	−0.004	0.010	0.737	1.000
Amino acid derivatives	2-ketobutyrate	1.07	−0.111	0.012	<0.0001	<0.0001
Amino acid derivatives	3-Methyl-2oxovalericacid	1.08	−0.117	0.012	<0.0001	<0.0001
Amino acid derivatives	Alpha-ketoisovalerate	1.14	−0.041	0.012	0.001	0.044
Amino acid derivatives	Dimethylglycine	2.93	−0.057	0.013	<0.0001	<0.0001
Amino acid derivatives	N-acetyl proteins	2.03	−0.055	0.014	<0.0001	0.003
Amino acid derivatives	Taurine	3.29	−0.042	0.012	<0.0001	0.027
Amino acid derivatives	Trimethylamine	2.87	−0.066	0.013	<0.0001	<0.0001
Amino acid derivatives	Trimethylamine-N-Oxide	3.25	0.027	0.013	0.042	1.000
Amino acids	Alanine	1.48	0.050	0.012	<0.0001	0.003
Amino acids	Arginine	1.71	−0.096	0.013	<0.0001	<0.0001
Amino acids	Asparagine	2.85	−0.087	0.014	<0.0001	<0.0001
Amino acids	Citrulline	3.14	−0.045	0.014	<0.0001	0.039
Amino acids	Glutamate	2.35	−0.038	0.013	0.004	0.171
Amino acids	Glutamine	2.45	−0.027	0.014	0.053	1.000
Amino acids	Glycine	3.57	−0.052	0.016	0.001	0.063
Amino acids	Histidine	7.06	−0.064	0.013	<0.0001	<0.0001
Amino acids	Lysine	1.76	−0.061	0.012	<0.0001	<0.0001
Amino acids	Phenylalanine	7.42	−0.038	0.012	0.002	0.093
Amino acids	Proline	3.34	0.017	0.014	0.199	1.000
Amino acids	Serine	3.96	−0.062	0.014	<0.0001	<0.0001
Amino acids	Threonine	4.29	−0.022	0.010	0.036	1.000
Amino acids	Tyrosine	7.2	0.008	0.013	0.503	1.000
Branched chain amino acids	Isoleucine	1.01	−0.099	0.013	<0.0001	<0.0001
Branched chain amino acids	Leucine	0.96	−0.144	0.011	<0.0001	<0.0001
Branched chain amino acids	Valine	1.05	−0.112	0.012	<0.0001	<0.0001
Glycolysis related	Citrate	2.66	−0.107	0.013	<0.0001	<0.0001
Glycolysis related	Glucose	3.25	0.109	0.013	<0.0001	<0.0001
Glycolysis related	Lactate	1.33	0.023	0.012	0.049	1.000
Ketone bodies	3-D-Hydroxybutyrate	1.18	−0.158	0.011	<0.0001	<0.0001
Ketone bodies	Acetate	1.91	−0.042	0.017	0.015	0.642
Ketone bodies	Acetoacetate	2.29	−0.112	0.009	<0.0001	<0.0001
Lipids	Lipid (mainly HDL, fatty acid CH_3_ moieties)	0.83	−0.053	0.015	<0.0001	0.016
Lipids	Lipid (mainly HDL, fatty acid (CH_2_)_n_ moieties)	1.23	−0.004	0.013	0.74	1.000
Lipids	Lipid (mainly LDL, fatty acid CH_3_ moieties)	0.87	0.063	0.014	<0.0001	0.001
Lipids	Lipid (mainly LDL, fatty acid (CH_2_)_n_ moieties)	1.27	0.060	0.014	<0.0001	0.001
Lipids	Lipid (mainly VLDL, fatty acid (CH_2_) moieties)	1.5	0.062	0.014	<0.0001	0.001
Lipids	Lipid ^1^H signal	5.18–5.21	0.031	0.015	0.037	1.000
Lipids	Phosphocholine containing lipids	3.21	−0.067	0.016	<0.0001	0.001
Organic acid	Creatine	3.93	−0.088	0.016	<0.0001	<0.0001
Organic acid	Creatinine	4.05	−0.060	0.014	<0.0001	<0.0001
Organic acid	Formate	8.45	−0.064	0.013	<0.0001	<0.0001

*Coef, coefficient indicating the directions of the associations between the metabolite and fasting glycemia overtime; SE, standard error for the coefficient*.

Of note, the analysis described positive associations of alanine and lactate with fasting glucose. In addition, the LDL and VLDL-related blood lipid signature was positively associated with fasting glucose concentrations throughout childhood. Most other amino acid metabolites, HDL and phosphocholine-related lipids were negatively associated with fasting glucose throughout childhood. The analysis also described how blood ketone bodies (3-D-hydroxybutyrate, acetoacetate), Krebs cycle intermediates (citrate, formate), glycine-related metabolites (dimethylglycine, creatine, creatinine) were negatively associated with the fasting glucose trajectories.

### Age Dependent Correlation of Blood Metabolites With Fasting Glucose

**Figure 2:**
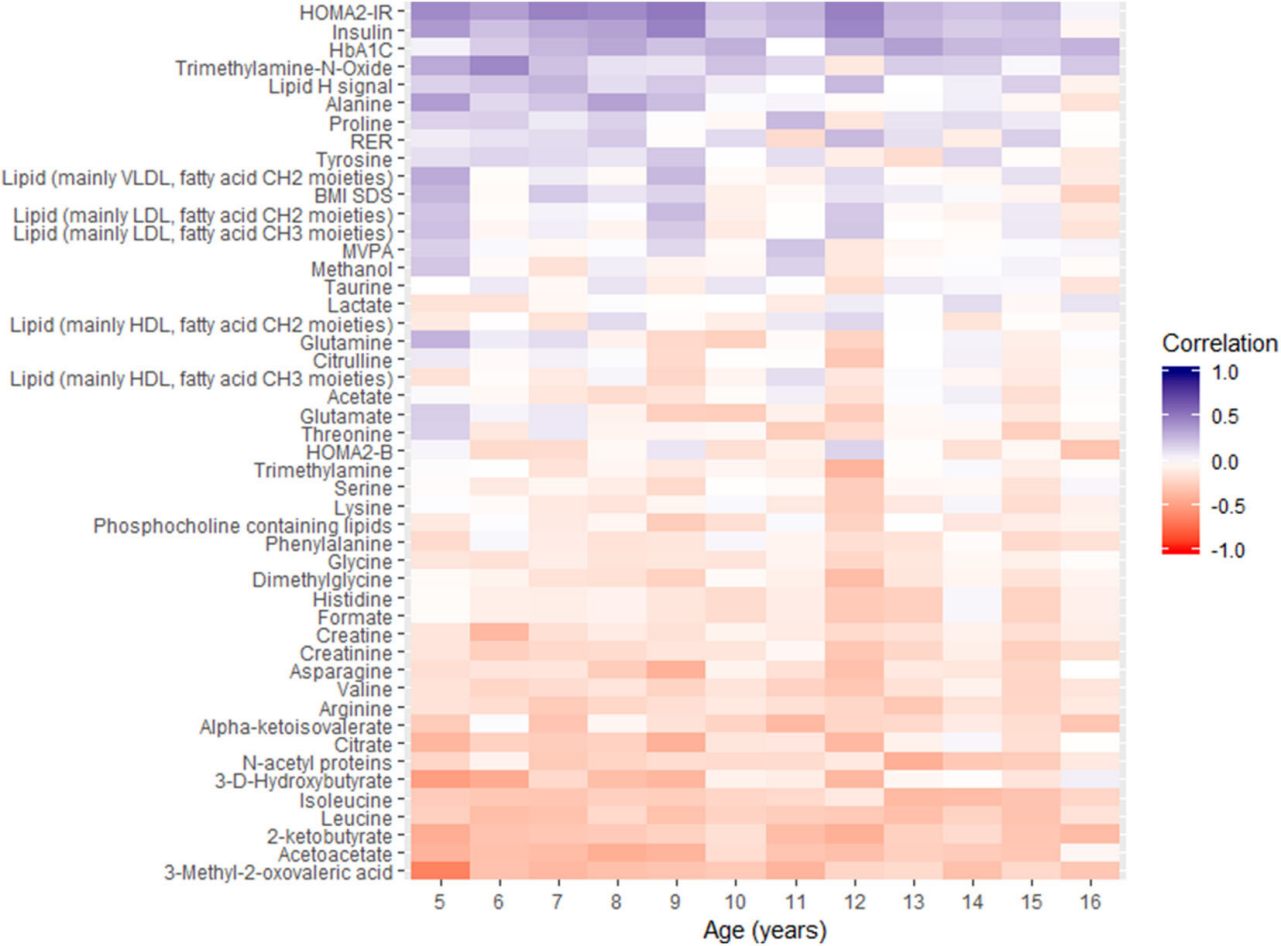
Overview of spearman correlations between fasting glucose and other parameters as a function of chronological age. MVPA, moderate to vigorous physical activity; RER, respiratory exchange ratio.

Additional cross-sectional correlation analysis of metabolites, insulin traits, HbA1c, respiratory exchange ratio, and BMI-z-scores with fasting glucose for each year was conducted using Spearman rank correlation. Data for the 12-year period were reported using heatmaps in Figure 2, for which the variables are ordered according to the temporal profile of their correlation with fasting glucose.

The heatmap plot highlights the negative correlations of α-keto-isovalerate, 3-methyl-2-oxovalerate, 2-ketobutyrate, 3-D-hydroxybutyrate, acetoacetate, citrate, and leucine with fasting glucose at each age, throughout childhood. In addition, positive associations of glucose with alanine, lactate, LDL, and VLDL related blood lipids were observed in the early years, between 5 and 9 years of age.

The same correlation analysis using HbA1c as endpoint variable was also performed and reported in Supplementary Figure 2. Fasting glucose and HbA1c showed positive correlations during childhood, and similar correlation patterns are observed between metabolites and fasting glucose and HbA1c. Yet, fasting glucose shows stronger statistically significant correlation with metabolites, and with insulin and insulin resistance, than HbA1c during childhood.

### Metabolite Changes According to Pubertal Stages and Metabolic Pathways

**Figure 3:**
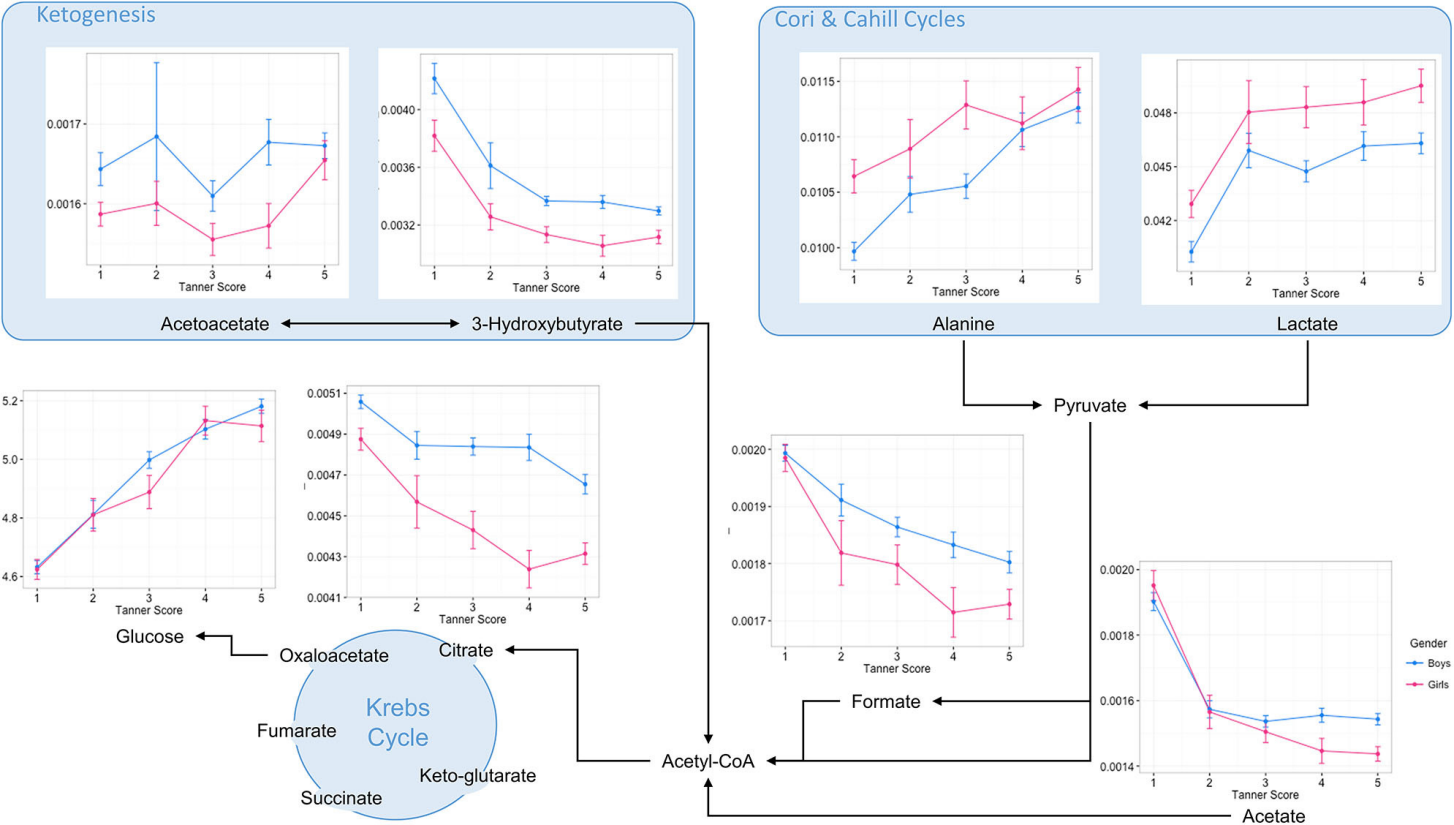
Overview of central energy metabolic pathways with selected blood serum metabolite patterns according to pubertal stages. Data are plotted as mean ± standard error of metabolite integrals (arbitrary units) and glucose concentration (Mmol/L) measured at the first occurrence of pubertal stages for boys and girls, respectively.

Major changes in levels of blood serum metabolites suggested changes in protein and amino acid levels, as well as lipid metabolic pathways. Therefore, blood biochemical patterns involved in central carbon metabolism, branched chain amino acids (BCAA), fatty acid oxidation, and ketogenesis were displayed according to their respective metabolic pathways (Figure 3, Supplementary Figures 3–5). Data are reported as a function of the pubertal stages for boys and girls.

Such a data visualization illustrates a rapid decrease in the level of ketogenesis from the early pubertal stages. These changes were associated with decreased levels of acetate, formate and the major Krebs cycle intermediate citrate, and are indicative of a profound remodeling of fatty acid oxidation in children's metabolism during the transition from early childhood to adolescence. In contrast to the changes in lipid metabolism, glucose and alanine concentrations increased steadily during puberty, whilst lactate concentration increased primarily in the early period of pubertal development. Such variations in blood biochemical profiles probably reflect changes in energy and carbohydrate metabolism during puberty, with alanine and lactate concentrations reflecting changing activity in the Cori and Cahill cycles.

Throughout puberty, changes in amino acid metabolism are more complex. Overall, children show a decreased blood concentration of several compounds, including glutamate, arginine, and glycine. In addition, complex patterns in the metabolism of branched amino acids (BCAA) are described. Whilst circulating levels of BCAA catabolic products decreased during puberty, circulating levels of BCAA evolved differently, and seemed to exhibit sexual dimorphism (e.g., Valine) (Supplementary Figure 3). Of note, several other blood amino acid profiles displayed distinct differences between boys and girls in late puberty. For instance, boys showed a distinct increase in glutamine and proline in late puberty, whilst girls showed decreases in histidine, asparagine, and citrulline (Supplementary Figure 4). Finally, creatinine metabolism shows a consistent pattern throughout puberty, with creatinine concentrations increasing steadily, and more markedly in boys from mid-puberty (Supplementary Figure 5).

## Discussion

As children grow and develop, changes in metabolism are directly related to total energy requirements (e.g., basal metabolism, physical activity, and growth) (26). Growth and development are associated with complex endocrine changes. In particular, the growth hormone (GH)/insulin-like growth factor 1 (IGF-1) axis has a fundamental impact on glucose homeostasis and metabolism throughout childhood, by influencing glycogenolysis, gluconeogenesis, and lipolysis (27). This description of puberty-related changes in molecular processes and substrate utilization for energy production significantly extends the existing literature. Although HbA1c retains a positive association with glucose throughout childhood in our cohort, it is weak, and their trends diverge from 10 years (16). These findings therefore limit the interpretation of HbA1c for the diagnosis of impaired fasting glycemia during childhood and suggest that factors other than glycaemia systematically influence the variance of HbA1c in youth (16). Our additional study reveals stronger associations of fasting glycemia with changes in insulin resistance as well as metabolites when compared to HbA1c, which suggests that analysis of temporal glycemic variations may encapsulate more comprehensively the changes in physiological and metabolite pathways during childhood. In this uniquely well-characterized cohort of healthy children, the transition from childhood to adolescence was associated with increasing fasting glucose concentrations and a complex remodeling of central energy metabolism, including amino acid and fatty acid molecular pathways.

In the EarlyBird cohort, the gradual rise in the fasting respiratory exchange ratio describes an increased carbohydrate oxidation throughout childhood. Yet, these fasting respiratory exchange ratio values are high in comparison to adults, where fasting respiratory exchange ratio would remain between 0.8 and 0.90 (28). Higher fasting respiratory exchange ratio values in adults (29) and in adolescents (30) may be linked to reduced metabolic flexibility (i.e., reduced ability to switch from fat to carbohydrate oxidation). Whilst there is limited published literature on healthy children, in the Earlybird cohort, we did not see statistically significant differences in fasting respiratory exchange ratio between normoglycemic children and those with impaired fasting glycemia. Since the maximum values are observed around 11–13 years of age, a period of height growth spurt and important growth in lean mass tissues, our observations may suggest a period of reduced metabolic flexibility during puberty. Finally, a potential limitation in the interpretation of the respiratory exchange ratio is that the measurements were conducted in the fasted state, and conclusions should not necessarily be extrapolated to the post-prandial state.

Prior to puberty, we identified that pre-pubertal children oxidize more fat relative to total energy expenditure than adults and pubertal children, an observation consistent with previous reports (31). In addition, pre-pubertal children are known to oxidize fats preferentially over carbohydrates during low to moderate intensity exercise as well, when compared with post-pubertal children and adults (32–35). Boisseau et al. reported that higher fat oxidation in pre-pubertal children was associated with a distinctive metabolic phenotype, namely increased blood free fatty acid and glycerol, which are indicators of fat mobilization from peripheral stores and increased lipolysis (35). Our study has also shown that pre-pubertal children have higher levels of ketogenesis, as noted by higher serum levels of ketones. Two ketone bodies, namely 3-D-hydroxybutyrate and acetoacetate, decreased linearly during the first two pubertal stages for both sexes, to reach minima that remained constant throughout the rest of childhood. Ketogenesis is generally stimulated when fatty acid β-oxidation and production of acetyl-CoA exceeds the processing capacity of the Krebs cycle. The decreased concentration of serum citrate and formate with puberty illustrates the decreased contribution of fatty acids to the pool of acetyl-coA entering the Krebs for energy production. These patterns describe an overall decreasing fatty acid oxidation, via β-oxidation and ketogenesis, from pre-pubertal to pubertal stage. Whereas 3-D-hydroxybutyrate showed the largest decrease in concentration, levels of acetoacetate remained more stable (constant levels), which suggests that there may be different contributions to ketogenesis from protein and lipid metabolism during puberty.

In addition, serum lipoprotein levels in childhood are known to vary with age, as a result of the hormonal changes of puberty, with reports of complex pattern and interactions according to age, gender and insulin resistance (36–38). Some studies in normal weight children reported that levels of triglycerides (mainly in VLDL) increased whereas total cholesterol and LDL-cholesterol decreased during puberty in both sexes (36, 37). Other reports describe distinct and gender-specific patterns from mid-puberty, namely increased triglycerides and decreased HDL cholesterol in boys, and the opposite pattern in girls (38). Our observations suggest that changes in the serum LDL and VLDL fatty acid signature are positively associated with fasting glycemia throughout childhood. We previously reported how IR development in the Earlybird cohort was marked by decreased phospholipids (mainly in HDL particles) and increased LDL fatty acid signature in both males and females in the EarlyBird cohort (15). Such an observation further illustrates the remodeling of lipid mobilization and metabolism that underpins structural growth and changing energy storage (36, 37).

As puberty commences and progresses, there are major changes in many physiological processes, which in turn modify fuel mobilization and utilization (39, 40). Jones and Kostyak reported higher fat oxidation in children (5–10 years) compared with adults—an adaptative process that might support normal growth requirements, such as higher rates of protein synthesis, lipid storage, and bone growth. Such higher requirements are captured in dietary recommendations for fat consumption, which suggest reduction in fat intake from childhood to adulthood (40, 41). For children 1–3 years of age, and 4–18 years of age, the Acceptable Macronutrient Distribution Range (AMDR) for total fat is 30–40% of energy, and 25–35% of energy, respectively (40, 41). In adults, the AMDR for fat has been set at 20–35% of energy (40, 41). The novel molecular insights into lipid metabolism before and during puberty, revealed in the present study, may help to further refine the dietary recommendations in terms of quantity and quality of lipids required for optimal growth and development of children before and during puberty.

Girls and boys are indistinguishable in muscle strength until puberty, at which time strength and aerobic performance increases more rapidly in boys (7, 20). Our analysis also revealed that serum creatinine increased from mid puberty more rapidly in boys than in girls, whilst being negatively correlated with fasting glucose. It is likely that the gender difference in muscle mass and function is driven primarily by the large difference in free testosterone concentrations that emerges with the onset of puberty (42). However, boys are more insulin sensitive than girls, especially during puberty, and it is possible that differences in the action of insulin may also contribute to gender difference in muscle mass and function. The gender-specific pattern of creatinine was associated with greater increases in serum leucine, valine, glutamine and proline in boys. Our observations agree with a recent report on whole blood amino acid patterns in puberty from the LIFE Child Cohort by Hirschel et al. (43). Serum creatinine is known to be affected by age, gender, ethnicity, dietary protein intake, and lean mass (44). During puberty, the bodies of boys exhibit a different tempo of bone, muscle, and cartilage/tendon growth, which is reflected in the gender-specific patterns of creatinine and amino acids. Amino acids play a major role as building blocks for protein synthesis and as regulators of key metabolic pathways for cell maintenance and growth (45). Previous studies reported that during puberty, growth is driven by maintaining a greater rate of protein synthesis than that of breakdown (46, 47). Arslanian et al. described lower protein oxidation and proteolysis during puberty when compared to pre-puberty, whereas protein synthesis was unchanged (46). In addition, they showed that during puberty whole body proteolysis is resistant to suppression by insulin (46). Blood amino acid concentrations reflect both the availability of amino acids and changes in amino acid influx or efflux between muscle and other tissues as a result of their utilization (e.g., by protein synthesis) or catabolism (protein turnover) (48, 49). In particular, proline, alanine, and glutamine are used as a source of energy metabolism through the anaplerotic pathway of the Krebs cycle in skeletal muscle (50). Since the efficiency of carbohydrate oxidation increases during puberty, we may hypothesize that increasing glycolytic metabolism reduces the mobilization of these amino acids into the anaplerotic pathway, and further contributes to higher circulating concentrations. The observed elevation of blood lactate and alanine concentrations with age reflects changes in the Cori and Cahill cycles. Since Cori and Cahill cycle shuttle lactate and alanine from the muscles to the liver, where the nitrogen enters the urea cycle for gluconeogenesis, this phenotype further illustrates the pubertal changes in glycolytic metabolism.

Last, several metabolites of one-carbon metabolism—glycine, dimethylglycine and creatine—showed a negative association with fasting glucose trajectories. This transmethylation pathway closely interconnects choline, betaine and homocysteine metabolism, and is of major importance for numerous cellular functions, such as DNA methylation, phosphatidylcholine, and protein synthesis (51, 52). Previous reports described how glycine and dimethylglycine metabolism is linked to glucose homeostasis and diabetes and may be genetically determined (53). In particular, lower circulating levels were associated with lower insulin sensitivity and higher fasting glucose (53), which is in agreement with our novel observations. With a potential role of the one-carbon cycle in the developmental origins of T2D (54), the biological implication of such a signature in the course of childhood would benefit from further clinical investigations.

It is recognized that there are several potential limitations with the present study. Importantly, the sample size was limited, and being an exploratory study, it was not possible to undertake an a priori power calculation. Furthermore, while less-invasive methods for measuring IR, such as the HOMA are well-suited for repeat measurements in cohort studies of children, it is recognized that a potential limitation is that IR measured by HOMA correlates only modestly with clamp-derived measures of IR, and also that HOMA IR already correlates highly with fasting insulin in normoglycaemic subjects (55, 56). However, if fasting insulin secretion is impaired, the direction of error is that HOMA underestimates IR. Despite these acknowledged limitations, HOMA is considered as a valid method for measuring IR in pediatric research (57).

## Conclusion

This study demonstrates that normal pubertal growth and development is accompanied by complex and extensive remodeling of metabolism and fuel oxidation, reflecting the changing energy requirements of puberty. The full complexity of this process is revealed by blood metabolic profiling. Fasting glycemia increases steadily throughout childhood and is accompanied by increasing concentration of insulin and rising respiratory exchange ratio. These metabolic changes are influenced by the endocrine changes of puberty, including the GH/IGF axis. As a result, the fuel economy shifts away from fatty acid oxidation and toward carbohydrate oxidation. The metabolic signatures indicate reduced fatty acid oxidation and ketogenesis, increased flux through Cori and Cahill cycles, and complex changes in amino acids with gender differences reflecting the emerging contrasts in body composition. There are gradual rises in LDL and VLDL particles and remodeling of one carbon metabolism. All of these changes represent normal physiological development.

These findings raise the important question at what point do physiological changes, such as increasing fasting glycemia begin to have pathophysiological consequences and raise concern for future cardiometabolic health? It is possible to speculate that the metabolic changes we have observed, especially the shift away from fat oxidation, and reduced ketogenesis, is maladaptive in the context of obesity, and may also be liable to perpetuate the obese state. Therefore, the reduced metabolic flexibility of puberty makes this a vulnerable period for excessive weight gain. Weight gain and obesity further exacerbate the physiological insulin resistance of puberty and fasting glycemia, and will favor atherogenic changes in the lipid profile and pathways, such as one carbon metabolism. This is in line with our other findings which suggested that weight gain and increasing insulin resistance will exacerbate hyperglycaemia (15) in adolescence, especially in those who also have genetic impairment of pancreatic beta cell function (13, 15).

Finally, these findings will have implications for guidance on child nutrition. Since fat, protein, and carbohydrate requirements change during pubertal development, this study suggests that macronutrient requirements for optimum healthy growth and development and reduction in risk of cardiometabolic disease may need to take into account metabolic changes at puberty and gender differences. We speculate that increasing respiratory exchange ratio and reduced ketogenesis may justify reduction in dietary fat relative to carbohydrate at adolescence, in order to reduce the risks of weight gain and insulin resistance. This nutritional change might be necessary earlier in girls, reflecting their earlier onset of puberty and growth spurt. The avoidance of adolescent weight gain is also emphasized, in view of the maladaptive metabolic effects of insulin resistance, and in order to reduce long term cardiometabolic risks. Since growth and energy metabolism are dependent also on the presence of small quantities of several micronutrients, further analyses should explore the potential influence of key enzyme cofactors on metabonomic profiles and implications for cardiometabolic risk. This knowledge has the potential to open-up the development of new and age-specific strategies for the prevention of cardiometabolic disease in children, through more evidence-based guidance on lifestyle and personalized dietary interventions.

## Data Availability Statement

The datasets presented in this article are not readily available because subject in particular, to ethical and privacy considerations. Requests to access the datasets should be directed to jonathan.pinkney@plymouth.ac.uk and francois-pierre.martin@rd.nestle.com.

## Ethics Statement

The studies involving human participants were reviewed and approved by Plymouth Local Research Ethics Committee. Written informed consent to participate in this study was provided by the participants' legal guardian/next of kin.

## Author Contributions

F-PM designed the study. AJ and F-PM were involved in the acquisition of the data. OC, F-PM, JH, and JP contributed to the analysis, data interpretation, and drafted the manuscript. JP was guarantor of the work. All authors approved the final version.

## Conflict of Interest

The authors declare that this study received funding from Bright Future Trust, The Kirby Laing Foundation, Peninsula Medical Foundation, Diabetes UK, the EarlyBird Diabetes Trust, and Nestlé Research. Nestlé Research had the following involvement with the study: metabonomics data generation and analysis, interpretation of data, writing of this article and decision to submit it for publication. The other funders were not involved in the study design, collection, analysis, interpretation of data, the writing of this article or the decision to submit it for publication. JH, JP, and AJ are employees of Plymouth University Peninsula School of Medicine and Dentistry. F-PM and OC are employees of Nestlé Research. JH and AJ have received funding from Nestlé Research. The authors have no other dualities of interest to declare.
