# Supplementary material for: Contributions of Fat and Carbohydrate Metabolism to Glucose Homeostasis in Childhood Change With Age and Puberty: A 12-Years Cohort Study (EARLYBIRD 77)
Source: Front Nutr. 2020 Aug 28;7:139. doi: 10.3389/fnut.2020.00139 (PMC7483556; doi:10.3389/fnut.2020.00139)
Supplement: Supplementary file 1 [file Table_1.DOCX]

**Supplementary Table 1. Age distribution according to tanner score for boys**

|  | | | | | | |
| --- | --- | --- | --- | --- | --- | --- |
|  | | ageT1 | ageT2 | ageT3 | ageT4 | ageT5 |
| N | Valid | 104 | 104 | 99 | 75 | 33 |
|  | Missing | 1 | 1 | 6 | 30 | 72 |
| Mean | | 9.606 | 10.624 | 12.709 | 13.579 | 14.464 |
| Median | | 9.495 | 10.540 | 12.730 | 13.590 | 14.540 |
| Std. Deviation | | 1.0374 | 1.0259 | .9351 | .7484 | .4999 |
| Minimum | | 7.4 | 8.4 | 10.8 | 11.5 | 13.4 |
| Maximum | | 14.1 | 15.1 | 15.1 | 15.2 | 15.3 |
| Percentiles | 25 | 8.830 | 9.845 | 11.960 | 12.960 | 13.960 |
|  | 50 | 9.495 | 10.540 | 12.730 | 13.590 | 14.540 |
|  | 75 | 10.120 | 11.203 | 13.170 | 13.980 | 14.845 |
|  | | | | | | |

Legend: ‘valid’ = number of valid observations; ‘missing’ = number of missing observations.

**Supplementary Table 2. Age distribution according to tanner score for girls**

|  | | ageT1 | ageT2 | ageT3 | ageT4 | ageT5 |
| --- | --- | --- | --- | --- | --- | --- |
| N | Valid | 43 | 44 | 40 | 31 | 5 |
|  | Missing | 2 | 1 | 5 | 14 | 40 |
| Mean | | 9.892 | 10.995 | 12.496 | 13.648 | 14.834 |
| Median | | 9.890 | 10.845 | 12.545 | 13.810 | 14.860 |
| Std. Deviation | | 1.0240 | 1.1801 | 1.0352 | .9317 | .5270 |
| Minimum | | 7.6 | 8.6 | 10.3 | 11.3 | 14.1 |
| Maximum | | 11.7 | 15.1 | 14.8 | 15.1 | 15.5 |
| Percentiles | 25 | 8.990 | 10.060 | 11.780 | 12.940 | 14.355 |
|  | 50 | 9.890 | 10.845 | 12.545 | 13.810 | 14.860 |
|  | 75 | 10.810 | 11.823 | 13.030 | 14.080 | 15.300 |
|  | | | | | | |

Legend: ‘valid’ = number of valid observations; ‘missing’ = number of missing observations.
