# Supplementary figures and images for: Contributions of Fat and Carbohydrate Metabolism to Glucose Homeostasis in Childhood Change With Age and Puberty: A 12-Years Cohort Study (EARLYBIRD 77)
Source: Front Nutr. 2020 Aug 28;7:139. doi: 10.3389/fnut.2020.00139 (PMC7483556; doi:10.3389/fnut.2020.00139)

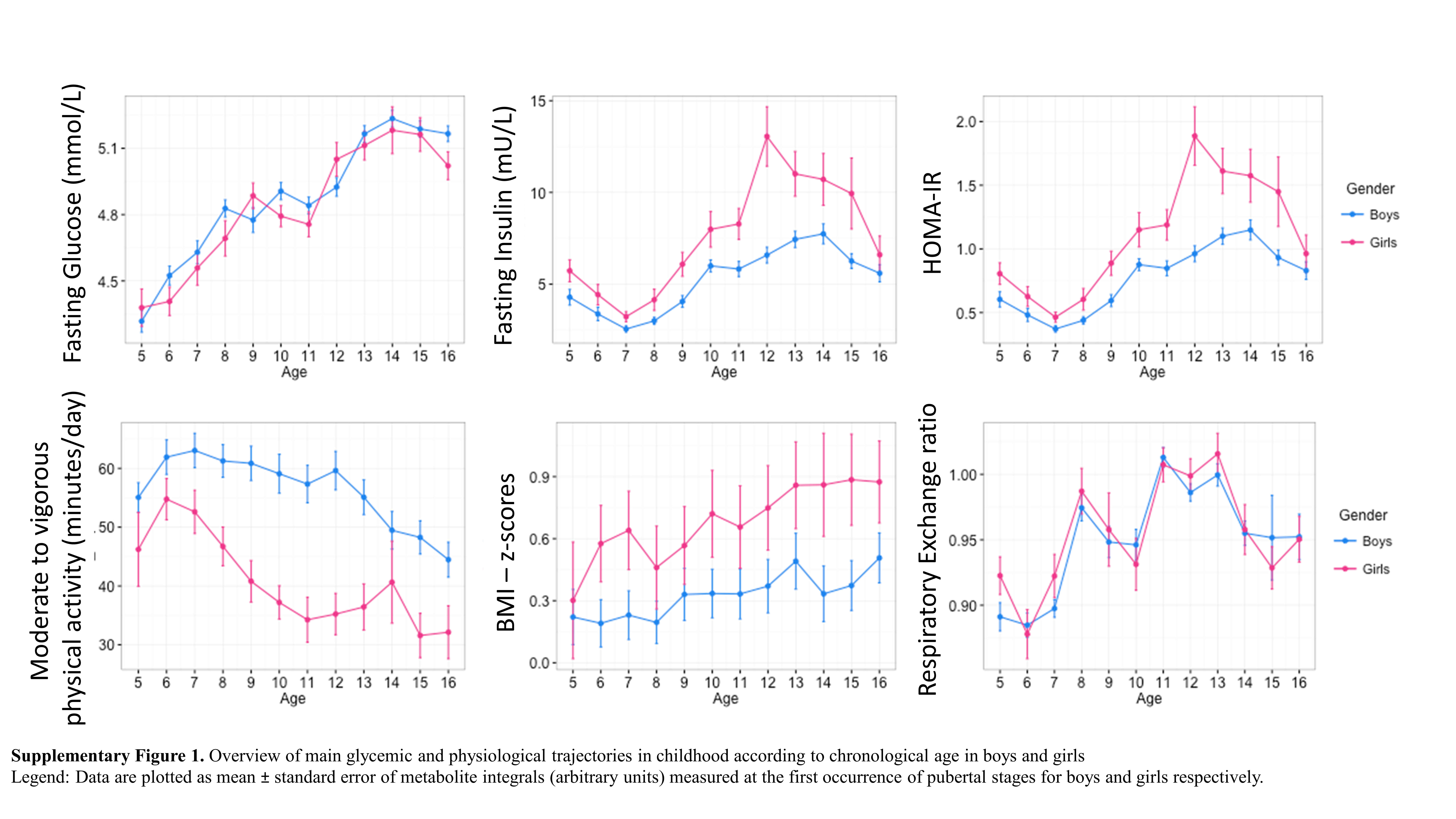

Supplement: Supplementary file 2 [file Image_1.tif]

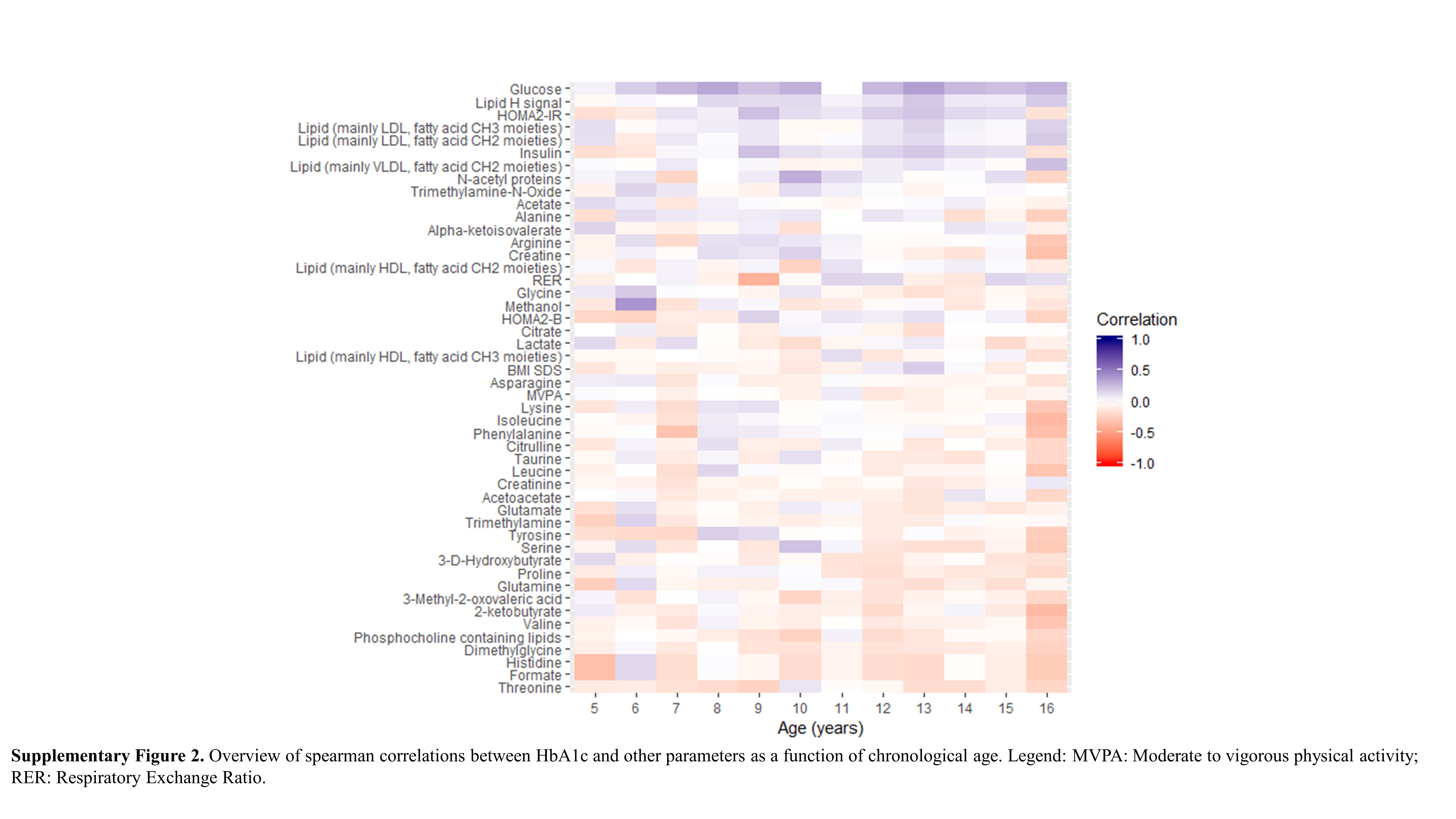

Supplement: Supplementary file 3 [file Image_2.tif]

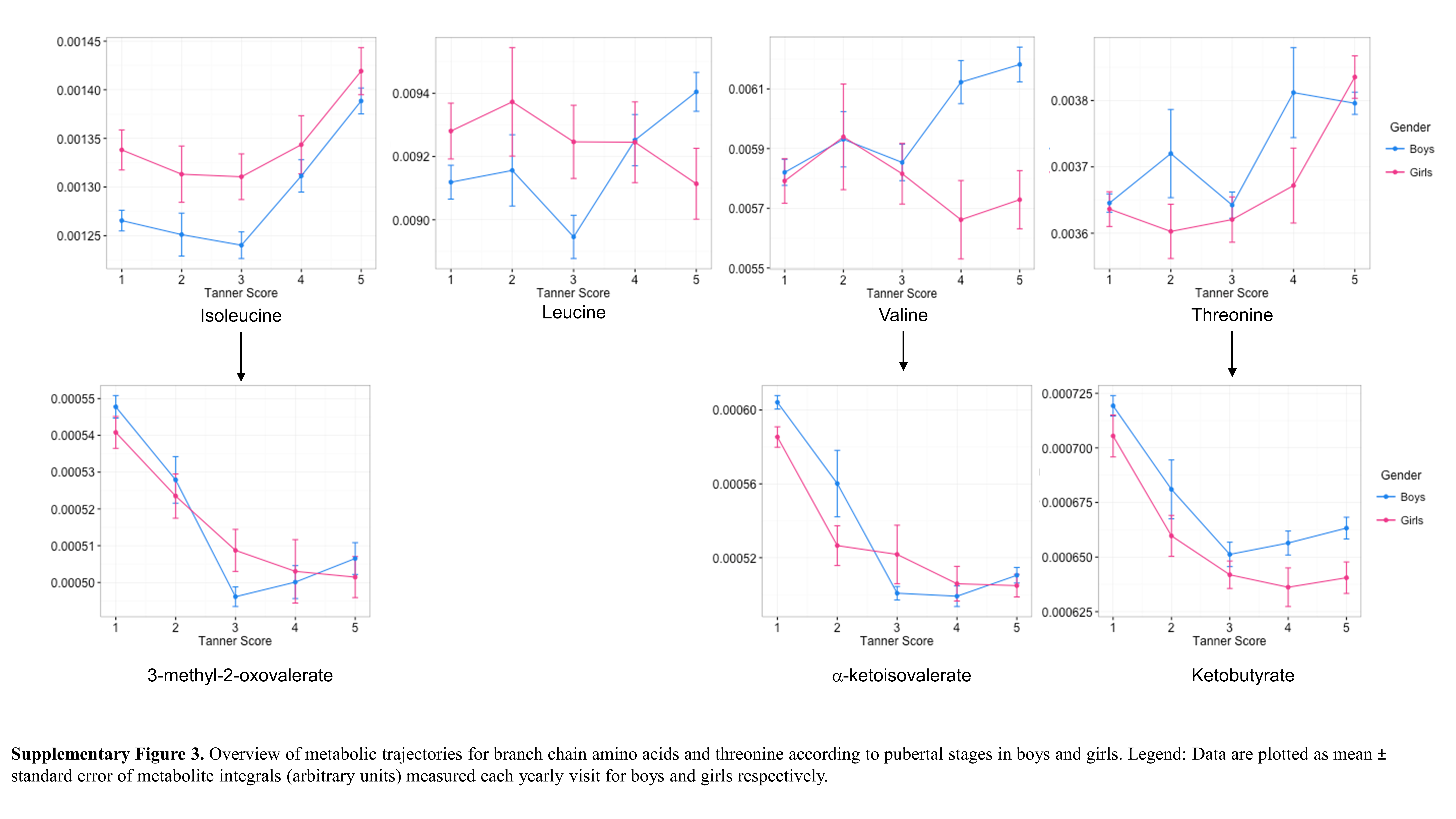

Supplement: Supplementary file 4 [file Image_3.tif]

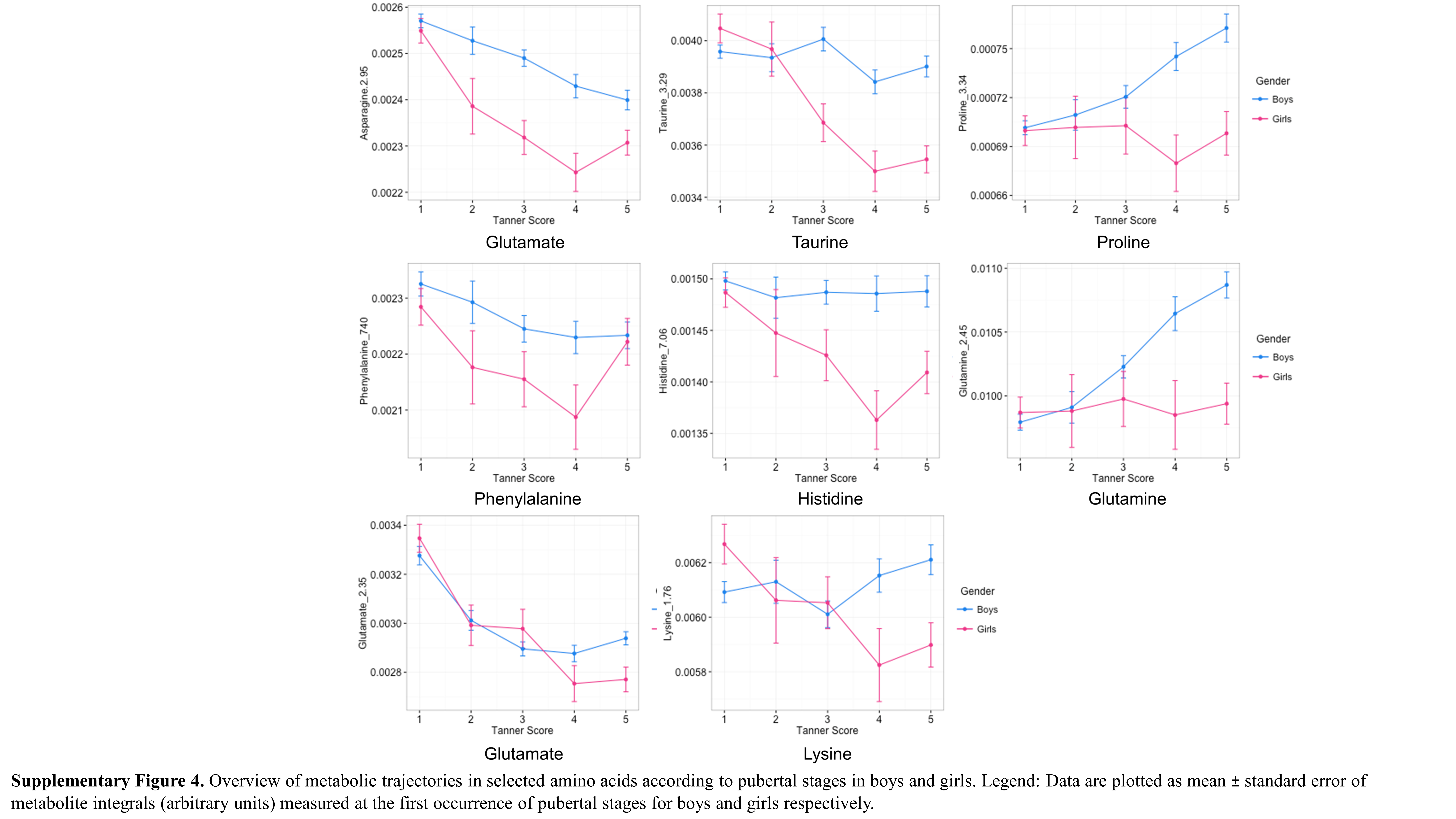

Supplement: Supplementary file 5 [file Image_4.tif]

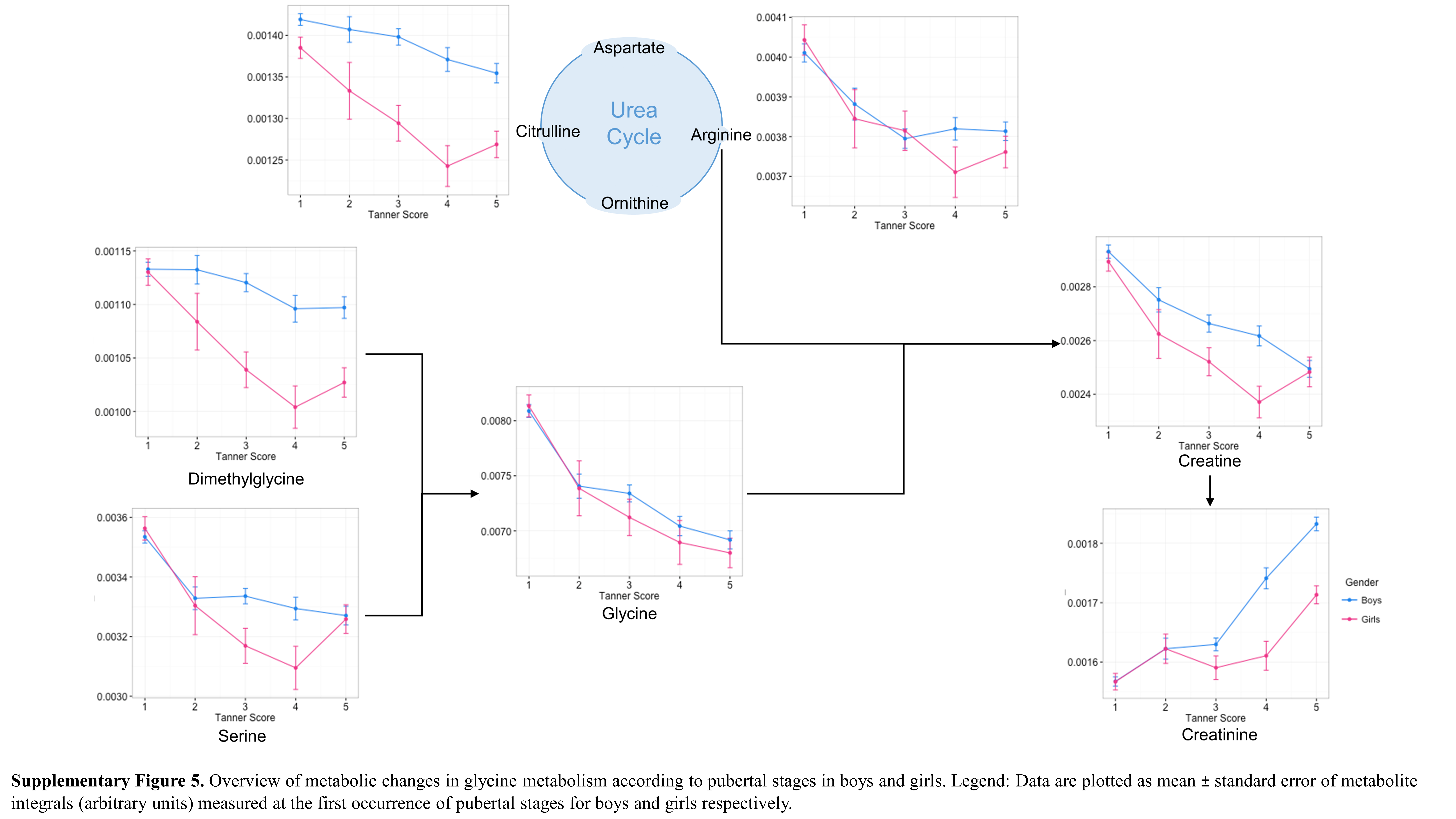

Supplement: Supplementary file 6 [file Image_5.tif]
